# Supplementary material for: Socio-economic deprivation and cancer incidence in England: Quantifying the role of smoking
Source: PLoS One. 2022 Sep 21;17(9):e0272202. doi: 10.1371/journal.pone.0272202 (PMC9491592; doi:10.1371/journal.pone.0272202)
Supplement: S1 File — (DOCX) [file pone.0272202.s001.docx]

**Socio-economic deprivation and cancer incidence in England: Quantifying the role of smoking**

**Supporting Information**

Nick W. S. Payne^1*^, Katrina F. Brown^1^, Christine Delon^1^, Yannis Kotrotsios^1^, Isabelle Soerjomataram^2^, Jon Shelton^1^

^1^ Policy, Information and Communication Directorate, Cancer Research UK, 2 Redman Place, London, E20 1JQ

^2^ Section of Cancer Surveillance, International Agency for Research on Cancer, Lyon, France.

* Correspondence: Nick Payne, [Nicholas.Payne@cancer.org.uk](mailto:Nicholas.Payne@cancer.org.uk)

S1 Table: ICD-10 codes and relative risks (RRs) for cancer types included in the analysis 2

S2 Appendix: Health Survey for England question phrasing for current smokers 4

S3 Table: Search terms for identifying relative risks5

S4 Appendix: Population Attributable Fraction formula for lung cancer second-hand smoke exposure6

S5 Appendix: Calculation of age-standardised incidence rates (ASR’s) by socio-economic position (deprivation quintile) for scenario 1 and scenario 2 in England 7

S6 Appendix: Calculation of deprivation-associated cancer cases for deprivation-related cancer types, smoking-related cancer types, scenario 1 and scenario 2 in England9

S7 Table: Population attributable fractions using all domains IMD for smoking prevalence by sex, age and deprivation quintile11

References 12

**S1 Table: ICD-10 codes and relative risks (RRs) for cancer types included in the analysis**

| **Cancer type** | **Deprivation-related** | **Smoking-related** | **Excess relative risk** | | | | |
| --- | --- | --- | --- | --- | --- | --- | --- |
|  |  |  | **Current** | | **Former** | | **Source** |
|  |  |  | **Females** | **Males** | **Females** | **Males** |  |
| Oral cavity (C00-C06) | Yes^a^ | Yes | 0.91 | 0.91 | 0.00 | 0.00 | Maasland et al.^[[1]](#endnote-1)^ |
| Salivary Glands (C07-C08) | Yes^a^ | No | - | - | - | - | - |
| Pharynx (C09-C10, C12-C14) | Yes^a^ | Yes | 2.43 | 2.43 | 0.00 | 0.00 | Gandini et al.^[[2]](#endnote-2)^ |
| Nasopharynx (C11) | Yes^a^ | Yes | 0.59 | 0.59 | 0.36 | 0.36 | Long et al.^[[3]](#endnote-3)^ |
| Nasal Cavity and Middle Ear (C30) | Yes^a^ | No | - | - | - | - | - |
| Accessory Sinuses (C31) | Yes^a^ | No | - | - | - | - | - |
| Larynx (C32) | Yes^a^ | Yes | 6.01 | 6.01 | 1.37 | 1.37 | Zuo et al.^[[4]](#endnote-4)^ |
| Oesophageal adenocarcinoma  (C15, ICD-O-3 814-838) | Yes^b^ | Yes | 1.32 | 1.32 | 0.62 | 0.62 | Tramacere et al.^[[5]](#endnote-5)^ |
| Oesophageal squamous cell carcinoma (C15, ICD-O-3 805-808) | Yes^b^ | Yes | 3.21 | 3.21 | 1.18 | 1.18 | Prabhu et al.^[[6]](#endnote-6)^ & Pandeya et al.^[[7]](#endnote-7)^ |
| Stomach (C16) | Yes | Yes | 0.30 | 0.63 | 0.00 | 0.42 | Li et al.^[[8]](#endnote-8)^ |
| Colorectal (C18-C20) | Yes | Yes | 0.11 | 011 | 0.15 | 0.15 | Cheng et al.^[[9]](#endnote-9)^ |
| Liver (C22) | Yes | Yes | 0.66 | 0.66 | 0.51 | 0.51 | Abel-Rahman et al.^[[10]](#endnote-10)^ |
| Pancreas (C25) | Yes | Yes | 0.90 | 0.90 | 0.20 | 0.20 | Lugo et al.^[[11]](#endnote-11)^ |
| Lung (C33-C34) | Yes | Yes | 5.99 | 6.33 | 2.13 | 2.14 | O’Keefe et al.^[[12]](#endnote-12)^ |
| Lung second-hand smoke | - | - | 0.41 | 0.41 | - | - | Jayes et al.^[[13]](#endnote-13)^ |
| Cervix uteri (C53) | Yes | Yes | 1.00 | *-* | 0.00 | *-* | Gandini et al.^2^ |
| Kidney (C64-C66, C68) | Yes | Yes | 0.27 | 0.57 | 0.20 | 0.29 | Liu et al.^[[14]](#endnote-14)^ |
| Bladder (C67) | Yes | Yes | 2.56 | 2.44 | 1.04 | 0.92 | van Osch et al.^[[15]](#endnote-15)^ |
| Small intestine (C17) | Yes | No | *-* | *-* | *-* | *-* | - |
| Anal (C21) | Yes | No | *-* | *-* | *-* | *-* | - |
| Gallbladder (C23) | Yes | No | *-* | *-* | *-* | *-* | - |
| Vulva (C51) | Yes | No | *-* | *-* | *-* | *-* | - |
| Vagina (C52) | Yes | No | *-* | *-* | *-* | *-* | - |
| Uterus (C54-C55) | Yes | No | *-* | *-* | *-* | *-* | - |
| Penis (C60) | Yes | No | *-* | *-* | *-* | *-* | - |
| Cancer of unknown primary  (C77-C80) | Yes | No | *-* | *-* | *-* | *-* | - |
| Hodgkin Lymphoma (C81) | Yes | No | *-* | *-* | *-* | *-* | - |
| Ovary mucinous  (ICD-10 C56-C57.4, ICD-O(3) 8480/3) | No | Yes | 0.44 | *-* | 0.00 | *-* | Santucci et al.^[[16]](#endnote-16)^ |
| Acute myeloid leukaemia  (C920, C924, C925, C926, C928, C930, C940, C942) | No | Yes | 0.52 | 0.52 | 0.45 | 0.45 | Colamesta et al.^[[17]](#endnote-17)^ |

a: Head and neck (C00-C14, C30-C32); b: Oesophagus (C15)

**S2 Appendix: Health Survey for England question phrasing for current smokers**

Current smoking prevalence in the Health Survey for England was assessed using the following phrasing:

For surveys in 2003^[[18]](#endnote-18)^, 2005^[[19]](#endnote-19)^ & 2007^[[20]](#endnote-20)^:

‘Do you smoke cigarettes at all nowadays’

For the survey in 2006^[[21]](#endnote-21)^:

‘Do you smoke cigarettes, cigars or a pipe at all these days’

**S3 Table: Search terms for identifying relative risks**

A smoking related search string was combined with smoking-related cancer types using AND. Searches were made in PubMed, and were supplemented using Google Scholar.

| **Risk factor** | **Search string** |
| --- | --- |
| Tobacco | tobacco OR cigarette OR smoking OR environmental tobacco smoke OR secondhand smoke |
| **Cancer Type** |  |
| Kidney cancer | (kidney OR renal OR renal cell) AND (cancer OR carcinoma OR tumour) |
| Bladder cancer | (bladder OR urothelium OR urothelial) AND (cancer OR carcinoma OR tumour) |
| Ovarian cancer | (ovary OR ovarian) AND (cancer OR carcinoma OR tumour) |
| Oral cavity cancer | (oral OR mouth) AND (cancer OR tumour) |
| Nasopharyngeal cancer | (nasopharynx OR nasopharyngeal) AND (cancer OR tumour) |
| Pharyngeal cancer | (oropharynx OR oropharyngeal OR pharynx OR pharyngeal) AND (cancer OR tumour) |
| Oesophageal cancer | (oesophagus OR oesophageal) AND (cancer OR adenocarcinoma OR squamous cell AND (cancer OR tumour) |
| Stomach cancer | (stomach OR gastric OR cardia) AND (cancer OR adenocarcinoma OR tumour) |
| Bowel cancer | (colorectum OR colorectal OR colon OR rectum OR rectal OR bowel) AND (cancer OR tumour) |
| Liver cancer | (liver OR hepatic OR hepatocellular) AND (cancer OR carcinoma OR tumour) |
| Pancreatic cancer | (pancreas OR pancreatic) AND (cancer OR adenocarcinoma OR tumour) |
| Laryngeal cancer | (larynx OR laryngeal) AND (cancer OR tumour) |
| Lung cancer | lung AND (cancer OR adenocarcinoma OR squamous cell carcinoma OR tumour) |
| Leukaemia (acute myeloid) | (leukaemia OR leukemia) AND myeloid |

**S4 Appendix: Population Attributable Fraction formula for lung cancer second-hand smoke exposure**

(p_1_ × ERR_1_) + (p_2_ × ERR_2_) + (p_3_ × ERR_3_)

----------------------------------------------------------------------

1 + [(p_1_ × ERR_1_) + (p_2_ × ERR_2_) + (p_3_ × ERR_3_)]

Where p_1_ is the proportion of ‘current cigarette smokers’ in England, p_2_ is the proportion of ‘ex-regular cigarette smokers’, p_3_ is the proportion of people exposed to second-hand smoke, ERR_1_ is the excess relative risk (relative risk – 1) for current smokers, ERR_2_ is the excess relative risk (relative risk – 1) for ex-smokers and ERR_3_ is the excess relative risk (relative risk – 1) for second-hand smoke exposure.

**S5 Appendix: Calculation of age-standardised incidence rates (ASR’s) by socio-economic position (deprivation quintile) for scenario 1 and scenario 2 in England**

Below is a key and corresponding pseudo R code used to calculate ASR’s by deprivation quintile.

**Key:**

CancerCases: number of cancer cases for smoking-related cancer types, scenario 1 and scenario 2 in England (2013-2017). For Scenario 1 and scenario 2, the incidence count was modelled and calculated as follows:

- Scenario 1: a = b-(c-d), whereby:

a: cancer cases for smoking-related cancer types if everyone had the same smoking prevalence as the least deprived

b: observed cancer cases for smoking-related cancer types

c: smoking attributable cancer cases for observed smoking prevalence

d: smoking attributable cancer cases if everyone had the same smoking prevalence as the least deprived

- Scenario 2: x = b – c, whereby:

x: cancer cases for smoking-related cancer types if nobody smoked

b: observed cancer cases for smoking-related cancer types

c: smoking attributable cancer cases for observed smoking prevalence

PopulationCount: population estimates for England (2013-2017).

AgeRange: 5-year age bands for observed smoking related cancer types. 5-year age bands for scenario 1 and scenario 2 between 0-24 years, then 10-year age bands for people aged 25+ because smoking prevalence data is presented in 10-year age bands which dictates the aggregation of the modelled incidence data.

Gender: male or female

IMD_quintile: socio-economic deprivation quintile

PopulationWeighting: 2013 European Standard Population Weight was applied by 5-year age bands for smoking-related cancer types. For scenario 1 and scenario 2, 2013 European Standard Population weight was applied by 5-year age band between 0-24 years, then 10-year age bands for people aged 25+ because smoking prevalence data is presented in 10-year aged bands, which dictates aggregation of the modelled incidence data.

**Code:**

# Join cancer incidence data with population data and 2013 European Standard Population Weighting for ASR calculation

asrSummary <- inner_join(CancerCases, PopulationCount, by=c("AgeRange", "Gender", "IMD_quintile"))

asrSummary <- inner_join(asrSummary, PopulationWeighting, by="AgeRange")

# ASR calculation

asrSummary <- asrSummary %>%

mutate(Numerator=ifelse(PopulationCount==0, NA, Cases*100,000 / PopulationCount*PopulationWeighting))

asr_quintile_sex <- asrSummary %>%

group_by(Gender, IMD_quintile) %>%

summarise(ASR = sum(Numerator) / sum(PopulationWeighting)) %>%

ungroup()

**S6 Appendix: Calculation of deprivation-associated cancer cases for deprivation-related cancer types, smoking-related cancer types, scenario 1 and scenario 2 in England**

Below is a key and corresponding pseudo R code used to calculate the deprivation-associated cases.

**Key:**

CancerCases: number of cancer cases for deprivation- and smoking-related cancer types, scenario 1 and scenario 2 (see appendix 1d for calculation of scenario 1 and scenario 2 cancer cases) in England (2013-2017).

PopulationCount: population estimates for England (2013-2017)

AgeRange: 5-year age bands for observed smoking related cancer types. 5-year age bands for scenario 1 and scenario 2 between 0-24 years, then 10-year age bands for people aged 25+ because smoking prevalence data is presented in 10-year age bands which dictates the aggregation of the modelled incidence data.

Gender: male or female

IMD_quintile: socio-economic deprivation quintile

AgeSpecificRate: age specific incidence rate by 5-year age band for deprivation- and smoking-related cancer types. 5-year age bands for scenario 1 and scenario 2 between 0-24 years, then 10-year age bands for people aged 25+ because smoking prevalence data is presented in 10-year age bands which dictates the aggregation of the modelled incidence data.

DepAssociatedCases: the number of excess cases due to incidence rates being higher in more deprived groups compared to the least deprived in England (2013-2017).

Quintile1_CancerCases: the number of ‘expected’ cancer cases if every deprivation group had the same incidence rate as the least deprived in England (2013-2017).

**Code:**

# Join data incidence data with population data for calculating rates and deprivation-associated cases

AgeSpecificSummary <- inner_join(CancerCases, PopulationCount, by=c("AgeRange", "Gender", "IMD_quintile"))

# Age specific rate calculation

AgeSpecificSummary <- AgeSpecificSummary %>%

group_by(Gender, AgeRange, IMD_quintile, PopulationCount) %>%

summarise(AgeSpecificRate = sum(CancerCases) / sum(PopulationCount)) %>%

ungroup()

# Excess case calculation

DepAssociatedCases <- AgeSpecificSummary %>%

group_by(Gender, AgeRange) %>%

mutate(Quntile1_Rate = AgeSpecificRate[which(IMD_quintile == 1)]) %>%

mutate(Quintile1_CancerCases = Quntile1_Rate * Population) %>%

mutate(ExcessCases = CancerCases – Quintile1_CancerCases %>%

ungroup()

# Sum excess cases by deprivation quintile

DepAssociatedCases_quintile_sex <- DepAssociatedCases %>%

group_by(Gender, IMD_quintile) %>%

summarise(yearlyExcess = sum(ExcessCases)) %>%

ungroup()

**S7 Table: Population attributable fractions using all domains IMD for smoking prevalence by sex, age and deprivation quintile**

| **Deprivation Quintile** | **25-64 years** | **65+ years** | **All ages (0-99 years)** |
| --- | --- | --- | --- |
| **Females** | | | |
| **1 (least)** | 4.9% | 9.9% | 7.9% |
| **2** | 6.0% | 11.6% | 9.4% |
| **3** | 7.9% | 13.2% | 11.1% |
| **4** | 10.1% | 17.4% | 14.3% |
| **5 (most)** | 14.2% | 22.0% | 18.4% |
| **Males** | | | |
| **1 (least)** | 9.2% | 13.3% | 12.1% |
| **2** | 11.5% | 15.3% | 14.1% |
| **3** | 14.5% | 18.0% | 16.8% |
| **4** | 17.0% | 21.3% | 19.7% |
| **5 (most)** | 23.6% | 25.2% | 24.3% |

**References**

1. Maasland, D.H.E., van den Brandt, P.A., Kremer, B. et al. Alcohol consumption, cigarette smoking and the risk of subtypes of head-neck cancer: results from the Netherlands Cohort Study. BMC Cancer **14**(1): 1-4 (2014), DOI: [10.1186/1471-2407-14-187](https://doi.org/10.1186/1471-2407-14-187) [↑](#endnote-ref-1)
2. Gandini, S., Botteri, E., Iodice, S. et al. Tobacco smoking and cancer: a meta-analysis. Int J Cancer **122**(1): 155-164 (2008), DOI: [10.1002/ijc.23033](https://doi.org/10.1002/ijc.23033) [↑](#endnote-ref-2)
3. Long, M., Fu, Z., Li, P. et al. Cigarette smoking and the risk of nasopharyngeal carcinoma: a meta-analysis of epidemiological studies. BMJ Open **7**(10): e016582 (2017), DOI: [10.1136/bmjopen-2017-016582](https://doi.org/10.1136/bmjopen-2017-016582) [↑](#endnote-ref-3)
4. Zuo, J.J., Tao, Z.Z., Chen, C. et al. Characteristics of cigarette smoking without alcohol consumption and laryngeal cancer: overall and time-risk relation. A meta-analysis of observational studies. Eur Arch Otorhinolaryngol **274**(3): 1617-1631 (2017), DOI: [10.1007/s00405-016-4390-x](https://doi.org/10.1007/s00405-016-4390-x) [↑](#endnote-ref-4)
5. Tramacere, I., Vecchia, C.L. & Negri, E. Tobacco smoking and oesophageal and gastric cardia adenocarcinoma: a meta-analysis. Epidemiology **22**(3): 344-349 (2011), DOI: [10.1097/EDE.0b013e31821092cd](https://doi.org/10.1097/ede.0b013e31821092cd) [↑](#endnote-ref-5)
6. Prabhu, A., Obi, K.O. & Rubenstein, J.H. Systematic review with meta-analysis: race-specific effects of alcohol and tobacco on the risk of oesophageal squamous cell carcinoma. Aliment Pharmacol Ther **38**(10): 1145-1155 (2013), DOI: [10.1111/apt.12514](https://doi.org/10.1111/apt.12514) [↑](#endnote-ref-6)
7. Pandeya, N., Williams, G.M., Sadhegi, S. et al. Associations of duration, intensity, and quantity of smoking with adenocarcinoma and squamous cell carcinoma of the oesophagus. Am J Epidemiol **168**(1): 105-114 (2008), DOI: [10.1093/aje/kwn091](https://doi.org/10.1093/aje/kwn091) [↑](#endnote-ref-7)
8. Li, W.Y., Han, Y., Xu, H.M. et al. Smoking status and subsequent gastric cancer risk in men compared with women: a meta-analysis of prospective observational studies. BMC Cancer **19**(1): 377 (2019), DOI: [10.1186/s12885-019-5601-9](https://doi.org/10.1186/s12885-019-5601-9) [↑](#endnote-ref-8)
9. Cheng, J., Chen, Y., Wang, X. et al. Meta-analysis of prospective cohort studies of cigarette smoking and the incidence of colon and rectal cancers. Eur J Cancer Prev **24**(1): 6-15 (2015), DOI: [10.1097/CEJ.0000000000000011](https://doi.org/10.1097/cej.0000000000000011) [↑](#endnote-ref-9)
10. Abdel-Rahman, O., Helbling, D., [Schöb](https://www.researchgate.net/scientific-contributions/Othmar-Schoeb-2111246687), O. et al. Cigarette smoking as a risk factor for the development of and mortality from hepatocellular carcinoma: An updated systematic review of 81 epidemiological studies. J Evid Based Med **10**(4): 245-254 (2017), DOI: [10.1111/jebm.12270](https://doi.org/10.1111/jebm.12270) [↑](#endnote-ref-10)
11. Lugo, A., Peveri, G., Bosetti, C. et al. Strong excess risk of pancreatic cancer for low frequency and duration of cigarette smoking: A comprehensive review and meta-analysis. Eur J Cancer **104**: 117-126 (2018), DOI: [10.1016/j.ejca.2018.09.007](https://doi.org/10.1016/j.ejca.2018.09.007) [↑](#endnote-ref-11)
12. O’Keefe, L.M., Taylor, G., Huxley, R.R. et al. Smoking as a risk factor for lung cancer in women and men: a systematic review and meta-analysis. BMJ Open **3**(10): e021611 (2018), DOI: [10.1136/bmjopen-2018-021611](https://doi.org/10.1136/bmjopen-2018-021611) [↑](#endnote-ref-12)
13. Jayes, L., Haslam, P.L., Gratziou, C.G. et al. SmokeHaz: Systematic Reviews and Meta-analyses of the Effects of Smoking on Respiratory Health. **150**(1): 164-179 (2016), DOI: [10.1016/j.chest.2016.03.060](https://doi.org/10.1016/j.chest.2016.03.060) [↑](#endnote-ref-13)
14. Liu, X., Peveri, G., Bosetti, C. et al. Dose-response relationships between cigarette smoking and kidney cancer: A systematic review and meta-analysis. Crit Rev Oncol Hematol **142**: 86-93 (2019), DOI: [10.1016/j.critrevonc.2019.07.019](https://doi.org/10.1016/j.critrevonc.2019.07.019) [↑](#endnote-ref-14)
15. van Osch, F.H.M., Jochems, S.H.J., van Schooten, F.J. et al. Quantified relations between exposure to tobacco smoking and bladder cancer risk: a meta-anlysis of 89 observational studies. Int J Epidemiology **45**(3): 857-870 (2016), DOI: [10.1093/ije/dyw044](https://doi.org/10.1093/ije/dyw044) [↑](#endnote-ref-15)
16. Santucci, C., Bosetti, C., Peveri, G. et al. Dose-risk relationships between cigarette smoking and ovarian cancer histotypes: a comprehensive meta-analysis. Cancer Causes Control **30**(9): 1023-1032 (2019), DOI: [10.1007/s10552-019-01198-8](https://doi.org/10.1007/s10552-019-01198-8) [↑](#endnote-ref-16)
17. Colamesta, V., D’aguanno, S., Breccia, M. et al. Do the smoking intensity and duration, the years since quitting, the methodological quality and the year of publication of the studies affect the results of the meta-analysis on cigarette smoking and Acute Myeloid Leukemia (AML) in adults? Crit Rev Oncol Hematol **99**: 376-388 (2016), DOI: [10.1016/j.critrevonc.2016.01.003](https://doi.org/10.1016/j.critrevonc.2016.01.003) [↑](#endnote-ref-17)
18. Health survey for England 2003. Methodology and documentation. Available from <https://webarchive.nationalarchives.gov.uk/ukgwa/20130107105354/http:/www.dh.gov.uk/prod_consum_dh/groups/dh_digitalassets/@dh/@en/documents/digitalasset/dh_4098912.pdf> [↑](#endnote-ref-18)
19. Health survey for England 2005. Methodology and documentation. Available from <https://files.digital.nhs.uk/publicationimport/pub01xxx/pub01184/heal-surv-heal-old-peo-eng-2005-apx-v1.pdf> [↑](#endnote-ref-19)
20. Health survey for England 2006. Methodology and documentation. Available from <https://files.digital.nhs.uk/publicationimport/pub01xxx/pub01213/heal-surv-cvd-risk-obes-ad-ch-eng-2006-apx-v4.pdf> [↑](#endnote-ref-20)
21. Health survey for England 2007. Methodology and documentation. Available from <https://files.digital.nhs.uk/publicationimport/pub00xxx/pub00415/heal-surv-life-know-atti-beha-eng-2007-rep-v3.pdf> [↑](#endnote-ref-21)
